# Continuous decline of hepatitis E virus seroprevalence in southern Germany despite increasing notifications, 2003–2015

**DOI:** 10.1038/s41426-018-0136-8

**Published:** 2018-07-25

**Authors:** Hannah Mahrt, Mathias Schemmerer, Gundula Behrens, Michael Leitzmann, Wolfgang Jilg, Jürgen J. Wenzel

**Affiliations:** 1 Institute of Clinical Microbiology and Hygiene, University Medical Center Regensburg, 93053 Regensburg, Germany; 2 Department of Epidemiology and Preventive Medicine, University of Regensburg, 93053 Regensburg, Germany

## Abstract

Hepatitis E virus (HEV) is viewed as an emerging pathogen. Many European countries, including Germany, have observed a steep increase of notified autochthonous hepatitis E cases in recent years. Our study investigated time trends in HEV seroprevalence in southern Germany between 2003 and 2015. A total of 3000 study sera were evenly distributed over sampling years 2003, 2006, 2009, 2012, and 2015, two age groups (20–29 and 30–39 years) and genders and were tested for anti-HEV IgG. Positive samples were quantified. The seroprevalence declined from 32.8% in 2003 over 22.5% in 2006 (*p* < 0.001) and 22.3% in 2009 to 17.7% and 17.8% in 2012 and 2015. A higher prevalence was found for males (*p* = 0.018) and the older age group (*p* < 0.001). Anti-HEV IgG concentrations ranged from 0.22 to 1783.19 WU mL^−1^. A higher median concentration (2.41 vs. 1.89 WU mL^−1^, *p* < 0.001) was found in the younger age group. The anti-HEV IgG seroprevalence decreased since 2003 and remains constant at ~18% since 2012. A rather low anti-HEV prevalence in young adults is indicative of a susceptible population and denotes a higher risk of HEV infections in this age group in the future. Therefore, reduction of HEV infection sources, close monitoring, and vigilance for proper control measures are warranted.

## Introduction

The hepatitis E virus (HEV) is a single-stranded, non-enveloped RNA virus identified in 1983^1^. This virus in the genus *Orthohepevirus* has increasingly attracted attention since 2008^2,3^. While there is only one known serotype, HEV genotypes differ by their epidemiology. Initially, HEV was associated with waterborne outbreaks in developing countries (genotypes 1 and 2). However, since 2008, an increasing number of foodborne zoonotic infections worldwide, caused by genotypes 3 and 4, have been reported.

The majority of HEV infections in Europe is reportedly caused by genotype 3 and is not travel associated. Known animal reservoirs include pigs, wild boars, and deer. Sources of infection are raw or undercooked meat as well as other contaminated food^4^. Moreover, transfusion-transmitted hepatitis E is well documented in several cases^5–7^. The vast majority of autochthonous HEV infections are usually asymptomatic and resolve without any known sequelae^8^.

There is a continuous increase of mainly autochthonous acute cases in Europe (e.g., 220 cases in 2010 vs. 1983 cases in 2016 in Germany; https://survstat.rki.de). However, the seroprevalence trends in various European countries are discordant. For example, a decrease in seroprevalence between 1991 and 2013 was reported for England^9^, Denmark^10^ and Germany^11,12^, while in the Netherlands an ~7% increase of anti-HEV IgG prevalence was reported between 2000 and 2010 among subjects aged 18–21 years^13,14^.

In this study, we hypothesize that if HEV infection is re-emerging in Europe, there should be an increase in seroprevalence especially in young adults, where a large proportion of subjects is seronegative and susceptible to infection. Therefore, we analyzed (i) whether there has been an increase in anti-HEV IgG prevalence among subjects aged 20–39 years in southern Germany between 2003 and 2015 and (ii) if anti-HEV IgG concentrations have changed or have shown age- or gender-specific variations over this time period. To address these questions, we qualitatively and quantitatively analyzed sera of 3000 individuals from 2003, 2006, 2009, 2012, and 2015 for the presence of anti-HEV IgG.

## Results

### Qualitative analysis

**Fig. 1:**
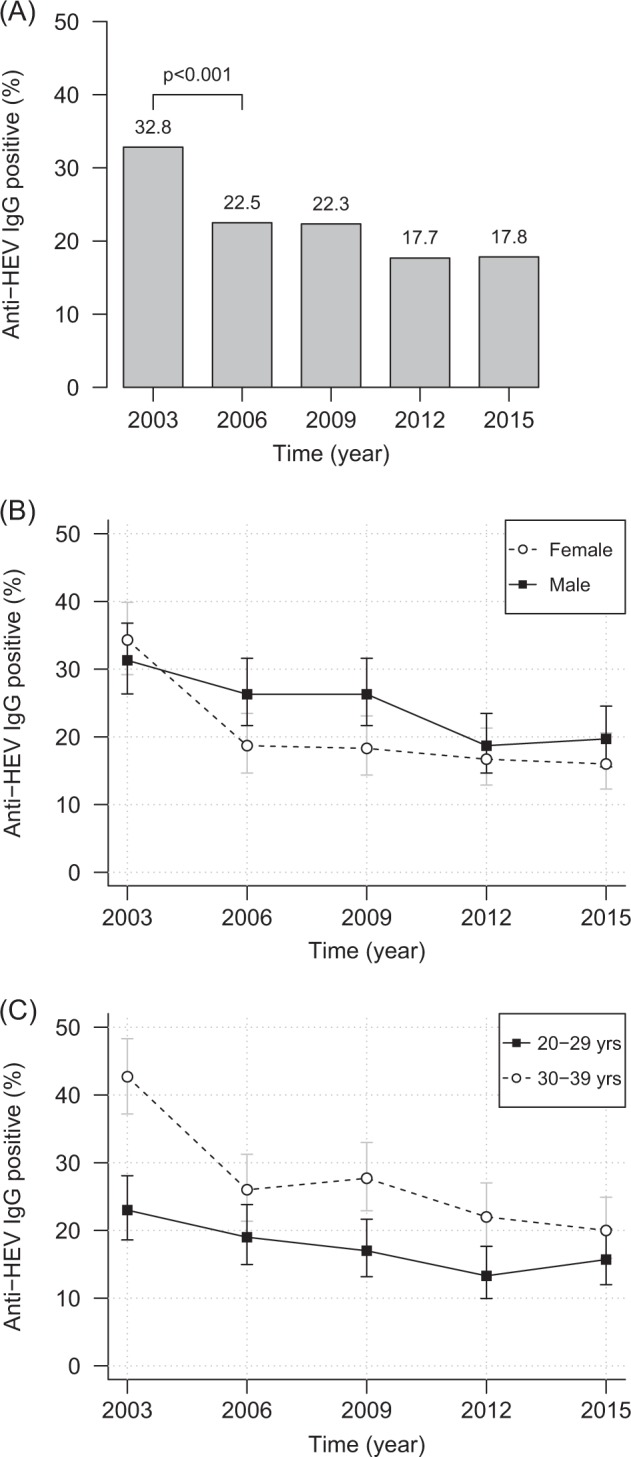
**Trend of anti-HEV IgG prevalence in 2003, 2006, 2009, 2012, and 2015.** **a** Overall prevalences. The chi square test with continuity correction was used to evaluate differences between the anti-HEV IgG prevalences of successive years. **b** Anti-HEV IgG prevalence and 95% CI divided by gender. **c** Anti-HEV IgG prevalence and 95% CI divided into age groups. Yrs, years

Our results show a time-dependent decrease of the anti-HEV IgG prevalence in the study population (Fig. 1a). The decrease started with 32.8% (2003) over 22.5% (2006) and 22.3% (2009), ended at 17.7% (2012) and remained constant at 17.8% in 2015. In total, a statistically significant (*p* < 0.001) decrease of 15.0% was observed when comparing 2003 and 2015.

When analyzing the sampling year groups according to age or gender, we observed that the prevalences in these groups mirrored the observed overall decrease (Fig. 1b, c). When comparing 2003 and 2015, the decrease was statistically significant for male (*p* = 0.001), female (*p* < 0.001), 20–29-year-old (*p* = 0.03), and 30–39-year-old (*p* < 0.001) subjects. The slight increase of prevalence in males as well as in the age group of 20–29 years between 2012 and 2015 was not statistically significant (*p* = 0.836 and *p* = 0.487) (Table 1).

**Table 1:** Anti-HEV IgG prevalence, overall, and by age and gender (percentages and absolute numbers of positives)

	2003	2006	2009	2012	2015
Overall	32.8% (197/600)	22.5% (135/600)	22.3% (134/600)	17.7% (106/600)	17.8% (107/600)
Gender					
Female	34.3% (103/300)	18.7% (56/300)	18.3% (55/300)	16.7% (50/300)	16.0% (48/300)
Male	31.3% (94/300)	26.3% (79/300)	26.3% (79/300)	18.7% (56/300)	19.7% (59/300)
Age group					
20–29 years	23.0% (69/300)	19.0% (57/300)	17.0% (51/300)	13.3% (40/300)	15.7% (47/300)
30–39 years	42.7% (128/300)	26.0% (78/300)	27.7% (83/300)	22.0% (66/300)	20.0% (60/300)

Apart from longitudinal changes, the anti-HEV IgG prevalence was significantly higher for men than women in 2006 and 2009 (*p* < 0.05). For the remaining years, there were no significant differences between gender-specific prevalences (Fig. 1b). Between 2003 and 2012, we observed statistically significantly higher prevalences for subjects aged 30–39 years than for the younger ones (2003: *p* < 0.001; 2006 borderline: *p* = 0.051; 2009: *p* = 0.002; 2012: *p* = 0.007). However, in 2015 there was no significant difference in prevalence between the age groups (Fig. 1c).

When analyzing additional subgroups (i.e., sampling year groups, further subdivided into gender-specific age groups; *n* = 150), the largest differences in prevalence were found between women aged 20–29 years and men aged 30–39 years: 2003 (23.3% vs. 40.0%, *p* = 0.003), 2006 (14.7% vs. 29.3%, *p* = 0.003), 2009 (15.3% vs. 34.0%, *p* < 0.001), and 2012 (11.3% vs. 22.0%, *p* = 0.02). Also, higher prevalences were identified for women aged 30–39 years compared to women aged 20–29 years in 2003 (*p* < 0.001) and 2012 (*p* = 0.02). Other subgroup-specific prevalences only differed marginally.

**Fig. 2:**
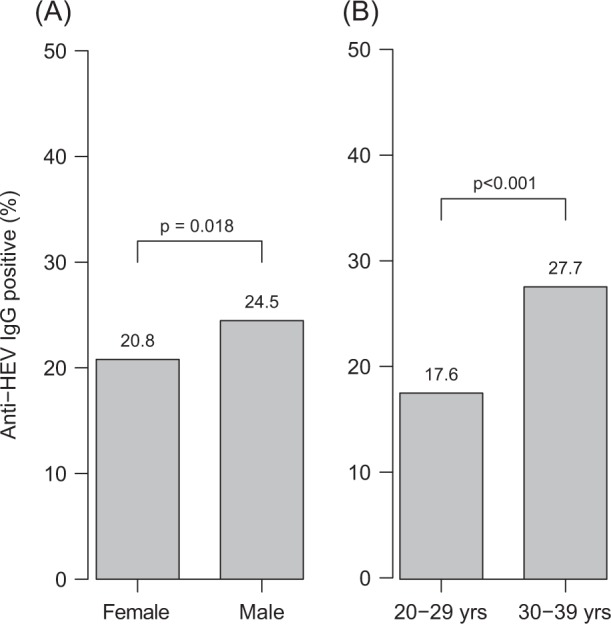
**Gender and age group analysis.** Anti-HEV IgG sorted by (**a**) gender and (**b**) age groups. Differences in prevalence were evaluated using the chi square test with continuity correction. Yrs, years

When considering only gender and age groups apart from sampling year groups, it became evident that male subjects were more often positive for anti-HEV IgG than females (24.5% vs. 20.8%, *p* = 0.018) (Fig. 2a). A similar observation was made for subjects aged 30–39 years in comparison to those aged 20–29 years (27.7% vs. 17.6%, *p* < 0.001) (Fig. 2b).

### Quantitative analysis

**Fig. 3:**
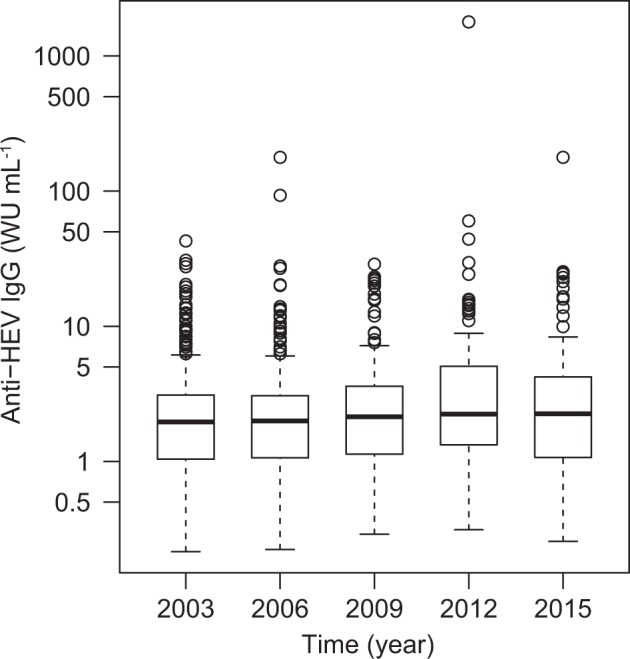
Distribution of anti-HEV IgG antibody concentrations by year of specimen collection. Data are shown as box-and-whisker diagrams. The box represents the interquartile range (IQR) between the 25th (Q1) and 75th (Q3) percentiles and includes a median line. Lower and upper whiskers represent Q1−1.5 × IQR and Q3 + 1.5 × IQR, respectively. Open data points show extreme outliers. WU, World Health Organization units

**Fig. 4:**
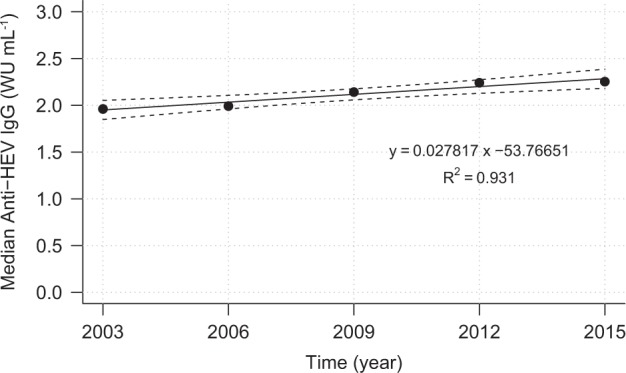
Linear regression model with 95% CI (dashed lines) for time-related development of median values of anti-HEV IgG antibody concentration. WU, World Health Organization units

The absolute concentration of anti-HEV IgG was determined in all specimens that tested positive (SCR ≥ 1). The concentration ranged from 0.22 to 178.05 WU mL^−1^, with the exception of one positive sample containing an exceptionally high concentration of 1783.19 WU mL^−1^ (a woman aged between 30 and 39 years, sampled in 2012). Figure 3 shows the distribution of concentration measurements for each sampling year as separate box-and-whisker diagrams. Figure 4 shows a linear regression model which was generated by fitting the median concentration values for each sampling year (1.96, 1.99, 2.14, 2.24, and 2.25 WU mL^−1^). However, the model only predicted a marginal overall annual increase of median anti-HEV IgG concentrations of about 0.03 WU mL^−1^ (Fig. 4).

The evaluation of the group-specific anti-HEV IgG concentrations showed that gender had no influence on the overall concentration level, regardless of the sampling year. In figures, the median value for males was 2.14 WU mL^−1^ compared to that of females with 2.06 WU mL^−1^ (*p* = 0.586). When regarding age groups, the median anti-HEV IgG concentration of subjects aged 20–29 years was significantly higher compared to subjects aged 30–39 years (2.41 vs. 1.89 WU mL^−1^, *p* < 0.001).

## Discussion

In this study, the development of anti-HEV IgG prevalence and concentration was investigated in adults aged 20–39 years between 2003 and 2015 in southern Germany. Our results demonstrate (i) a significant overall decrease of anti-HEV IgG-positive young adults, among which men and the upper age group (30–39 years) have the highest prevalence.

Quantitatively noticeable is (ii) a slightly increasing anti-HEV IgG trend with an overall median annual increase of 0.03 WU mL^−1^. It is also remarkable that younger persons have higher levels of antibodies than older individuals, while there is no gender-specific difference in antibody concentrations.

In a previous study, we found a decline of the HEV-antibody prevalence from 20.5% in 1996 to 14.5% in 2011 by immunoblot analysis in the same region of southern Germany^11^. In a recently published study, we reported very similar figures for the whole of Germany with 18.6% in 1998 and 15.3% in 2010^12^. Therefore, it is tempting to speculate that our current findings might also be generalizable for the whole of Germany. However, caution is advisable at this point since our study was not designed to assess the situation for the whole country and intentionally focused on subjects of 20–39 years of age. Comparability between studies is therefore limited since the former studies included subjects between 18 and 80 years. Moreover, there may be some geographic regions which do not follow the observed continuous decline, possibly due to regional or dietary factors, as has been reported for France^15^.

In Denmark, a comparable decrease of anti-HEV IgG prevalence was observed^10^. Using an in-house assay, a prevalence of 32.9% in 1983, 20.6% in 2003, and 10.7% in 2013 was found. In order to achieve more comparable data, the specimens tested in 2013 had been re-tested by the authors using the Wantai assay (which was also used in the present study). The prevalence by means of the Wantai assay was 19.8%, while all subjects which had been tested positive by the in-house assay were found positive by the Wantai assay as well. Although the absolute numbers are hardly comparable to the results found in our study, the time development seen in Denmark is also in line with our findings^10^. Using the Wantai assay, a comparable prevalence rate as well as a decrease over time was found in the US: 21.8% in 2006 and 16.0% in 2012^16^.

The higher prevalence in the upper age groups may be explained by the cumulative risk of infection over a subject’s lifetime. Multiple studies have consistently observed this phenomenon in several geographic regions^10–12,16,17^.

Surprisingly, we found men significantly more often infected with HEV than women in our study. In contrast, no correlation between anti-HEV IgG positivity and gender has been detected in other seroprevalence studies^10–12,16^. To our knowledge, this is the first time that a statistically significant gender-specific difference in anti-HEV IgG prevalence was observed. Among the various factors that may lead to a higher anti-HEV IgG prevalence in male subjects, we speculate that men more often engage in specific hobbies or professions that may pose an elevated HEV infection risk, such as hunting or veterinary medicine^18,19^. Moreover, men in Germany consume more pork than women^20^, which is considered the most important transmission route of HEV in industrialized countries^21^.

For the Netherlands it was reported that the prevalence of anti-HEV IgG in younger adults increased significantly (*p* = 0.016) from 4.3% (5/116) in 2000 to 12.7% (23/181) in 2011^13^. Our study investigated a comparable time period and study group in southern Germany and allowed us to exclude such an effect. One possible explanation for this difference is the variability in anti-HEV IgG prevalence between different European countries or even smaller geographic regions^15,22^.

Other authors mentioned differences in laboratory test characteristics as a possible reason for non-comparable and strongly varying HEV prevalence data across European countries^17,22–24^. In a previous study, we had also observed how differences in the analytic sensitivity of a broadly applied enzyme-linked immunosorbent assay (ELISA) as compared to an immunoblot resulted in differing seroprevalence estimates: for subjects between 20 and 70 years and sampled in 2011, we found 34.3% HEV IgG positive by the former and 14.5% by the latter^11^. Although such different test characteristics should not influence the ratio between groups tested at different time points with the same test, we decided to use the well characterized and highly sensitive Wantai ELISA in our study, that was additionally calibrated by using the WHO reference reagent^25^.

Quantitative analyses revealed an increasing level of anti-HEV IgG antibody concentrations over time. Although the overall annual increase of 0.03 WU mL^−1^ (1.59% of the median overall antibody level) was found to be statistically significant, it is very likely not of any clinical relevance.

The higher antibody concentrations detected in the lower age group of 20–29 years may reflect a higher proportion of subjects among all positives who have recently experienced the first HEV infection in their lifetime. Consequently, those subjects will likely have higher anti-HEV IgG concentrations as compared to those who had the infection in the more distant past since anti-HEV IgG generally decreases with time^26^. By comparison, gender had no influence on anti-HEV IgG antibody concentrations. Bendall et al.^27^ had found no relationship between HEV IgG concentration and (among other variables) gender. Zhang and coworkers had investigated geometric mean concentrations (GMC) of HEV antibodies in a study on subjects with naturally acquired and vaccine-induced immunity: GMC of seropositive subjects were stable among different age groups and between genders^28^.

Our findings are in contrast to the increasing number of notified acute HEV cases seen in many years. Hepatitis E is a notifiable disease in 17 of 28 European countries^29^. Four additional countries that lack a notification system have implemented surveillance through HEV reference laboratories. Since 2009, many countries observed a proverbial explosion of laboratory-confirmed autochthonous hepatitis E cases (2009–2014; Italy: +130%, Finland: +250%, Hungary: +350%, England & Wales: +500%, Germany: +615%, France: +875%)^29^. This common trend cannot be denied, although cautious interpretation is advisable due to highly variable surveillance systems and case definitions within the European Union. In Germany, HEV with clinical symptoms is notifiable as a laboratory-confirmed case, if (a) anti-HEV IgM is present, or if (b) an IgG increase in paired samples is detected, or if (c) HEV RNA is detected. Since there is no directive to confirm a serologically defined case by RNA detection, it is conceivable that part of the notified cases are actually older infections (IgM usually persists at least 4 months)^30^ or false-positive IgM results. In fact, only 40% of all samples with positive anti-HEV IgM detection are found positive by HEV RT-qPCR in the German HEV reference laboratory (own unpublished data). Hence, there is good reason to believe in at least some degree of over-reporting of acute HEV cases. Moreover, we speculate that the phenomenon of HEV being an emerging disease in Germany is most probably due to an increased awareness of hepatitis E as an autochthonous infection and due to the broader availability of improved serologic test systems.

The results of our study demonstrate a significant seroprevalence decrease in young adults. These findings indicate a strong reduction in HEV infection pressure over the last decades. For example, in 2003 and 2006, the younger age group in our study had virtually the same seroprevalence rate approximately 10 years later (23% vs. 22% and 19% vs. 20%, Table 1). This reduction in HEV infection risk is in line with results of a study on the evolutionary history and population dynamics of HEV: based on computational modeling, the authors propose that genotypes 3 and 4 experienced an increase in population size in the first half of the twentieth century, followed by a decline of unknown cause around 1990^31^. A second study from Japan points in the same direction. However, the respective model shows an increase in HEV genotype 3 population size around 1960–1970, followed by a decline around 2000^32^.

**Fig. 5:**
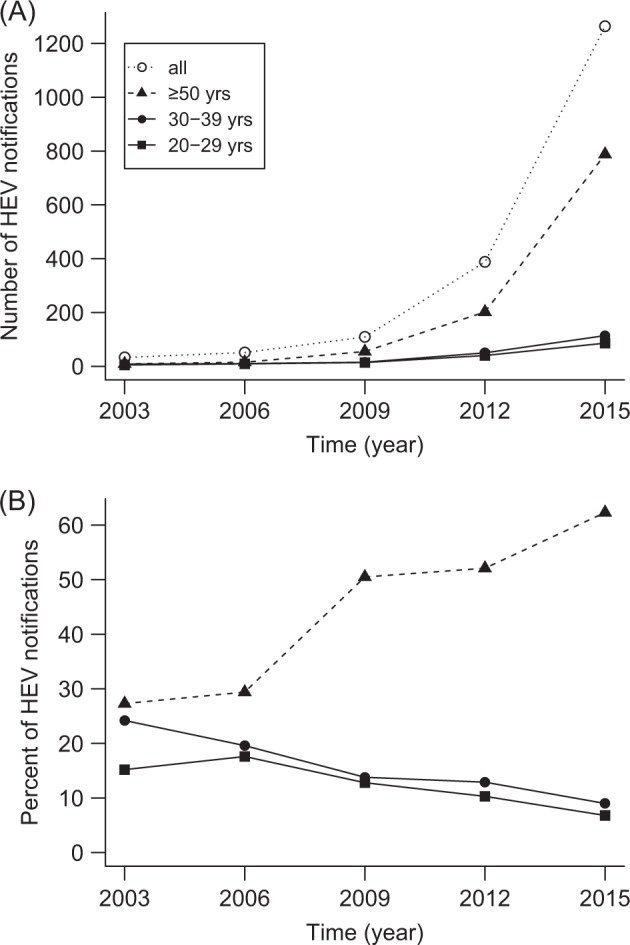
**Hepatitis E notifications in Germany.** (**a**) Absolute numbers and (**b**) percentage in three selected age cohorts, 2003–2015. Percentages are calculated in relation to all notified cases per year. Yrs years

The number of officially notified hepatitis E cases has increased considerably during the past decades in Germany. The respective absolute numbers reported between 2003 and 2015 are shown in Fig. 5a for three selected age groups (20–29, 30–39, and ≥ 50 years; data source: https://survstat.rki.de). The most prominent absolute increase was recorded in the older age group (≥50 years). Interestingly, the proportion of notified hepatitis E in subjects ≥50 years has also increased considerably, while the proportion of the age groups between 20–29 and 30–39 years has decreased continuously (Fig. 5b). A similar trend exists in the federal state of Bavaria, where the majority of our study samples was collected (data not shown). This can likely be explained by the decline in HEV infection pressure over the last decades, which results in a higher proportion of susceptible individuals and consecutively more notified acute cases in the older age group in which HEV infections seem to become more often symptomatic. This would also be in line with the observed lower seroincidence in young adults in the last decade. During the same period, the majority of notified cases were males (2003: 82%, 2006: 65%, 2009: 66%, 2012: 63%, 2015: 61%). For the first time in a seroprevalence study on HEV, we found this gender-specific effect in young adults aged 20–39 years. Both observations can be summarized as an increasing general trend for men and older people being reported with autochthonous HEV infections^33^.

It is a limitation of our study that we could only test left-over sera of confined geographic origin. Due to the study design we cannot exclude that some samples were derived from immunosuppressed individuals, which could marginally influence quantitative data. However, we assume that this will not notably bias the overall quantitative and qualitative results of the study. Also, the study specimens were not derived from a randomized study of the whole German population that would allow claiming prevalence trends for the entire country. Hence, the generalizability of our results may be limited.

In order to detect a potential selection bias, the origin of all samples was analyzed as to the referring medical specialization. No relevant variations were found over the years (data not shown), indicating that there were indeed no changes in the “drainage area” of our laboratory. Moreover, venipuncture is a frequent medical procedure associated with a wide variety of indications. The potential that a certain group of individuals was over-sampled, thereby potentially biasing the results, is low. Furthermore, we can exclude a response bias, because there was no need for the study subjects to react to a study invitation.

In summary, our study demonstrates a decreasing anti-HEV IgG prevalence over a decade in young adults. The prevalence has remained constant at around 18% since 2012. The median concentration of specific antibodies was practically the same throughout the investigated time period. A low anti-HEV prevalence in a population is a significant substrate of susceptible population and might be the “silence before a storm”: i.e. a higher rate of HEV infections, especially in young adults in the future. Therefore, more work is needed to elucidate and reduce HEV infection sources and reservoirs. Moreover, close monitoring, vigilance for proper control measures, and comparative studies to investigate the development of prevalence in different geographic regions will be of importance in such conditions.

## Materials and methods

### Study population

The study cohort consisted of 3000 subjects between 20 and 39 years of age with residence in southern Germany. Medical personnel were excluded in order to ensure that the study cohort represents an acceptable surrogate to the local general population. Apart from that, no criteria other than the donor’s age and date of collection were taken into account. Selection of specimens was not carried out according to any specific indication but was performed using a random sample from all available left-over sera as described below.

### Study design

The years 2003, 2006, 2009, 2012, and 2015 were chosen as time-series for the determination of anti-HEV seroprevalence. Since anti-HEV IgG prevalence increases with age^11,34,35^, only subjects between 20 and 39 years were included to better detect potential prevalence changes. Based on previous results of anti-HEV IgG antibody studies^11^, we assumed a prevalence rate of 13.4% in 2011 for the age group of 20–39 years. An annual prevalence increase of 0.5% was proposed as a working hypothesis^13^. Using the G*Power 3.1 software^36^, a sample size of 600 subjects per year was found to be necessary to detect a statistically significant effect with *α* = 0.05 and 95% statistical power.

After applying the exclusion criteria described above and after eliminating duplicate specimens, 15,082 available samples were sorted according to sampling year, age group (20–29 and 30–39 years), and gender. Subsequently, 150 samples per subgroup were randomly chosen using the *sample()* function implemented by the R programming language (R Foundation for Statistical Computing). By following this approach, a total of 3000 samples were selected.

### Sample collection

All study specimens were obtained as part of the daily routine operations of our diagnostic laboratory in 2003 through 2015. Only surplus serum stored at −20 °C was used for this study.

### Qualitative analysis

All samples were analyzed for anti-HEV IgG using a commercially available (Wantai, China). The assay was performed according to the manufacturer’s instructions. Washing steps were executed using the automated HydroFlex ELISA washer (TECAN, Austria). For interpretation of results, all specimens with a signal-to-cut-off ratio (SCR) ≥1 in single determination were considered positive. Samples with an SCR <1 were considered negative.

### Quantitative analysis

To determine absolute anti-HEV IgG concentrations and to adjust for potential product batch and interassay variations, we used the WHO reference reagent for antibodies to HEV (NIBSC code: 95/584). Calibration was done by performing an ELISA run with a twofold reference reagent dilution series. Anti-HEV IgG concentration started from 100 WHO Units per mL (WU mL^−1^) and was diluted in 20 steps to a final concentration of 1.91 × 10^−4^ WU mL^−1^. The measured values showed a typical sigmoid curve (data not shown).

The quantification range was between 0.10 and 15.79 SCR. Only positive samples (SCR ≥ 1) were considered. Specimens with an SCR above the quantification range were diluted and re-tested.

Individual calibrations of each ELISA run were implemented with four standardized WHO reference reagent dilutions covering the approximate quantification range (3.13, 0.78, 0.20, and 0.05 WU mL^−1^). The SCR values were converted by a linear regression model using SigmaPlot 12.3 (Systat Software Inc., USA) and Microsoft Excel (Microsoft Corp., USA).

### Statistical methods

Statistical data analysis was performed using IBM SPSS Statistics 23 (IBM Armonk, USA). For the evaluation of quantitative data, the Mann–Whitney *U* test was used and for the evaluation of qualitative data a chi-squared test with continuity correction was performed. A statistically significant difference was defined as *p* < 0.05. Confidence intervals (CI) of 95% were calculated with the Wilson score interval. Statistical regression analyses were performed using the R programming language (R Foundation for Statistical Computing).
